# Revue d’épidémies de grippe humaine avant la mise en place de la surveillance sentinelle en République Démocratique du Congo

**DOI:** 10.11604/pamj.2017.27.35.10197

**Published:** 2017-05-11

**Authors:** Edith Nkwembe-Ngabana, Steve Ahuka-Mundeke, Benoit Kebela-Ilunga, Emile Okitolo Londa, Jean-Jacques Muyembe-Tamfum

**Affiliations:** 1 Institut National de Recherches Biomédicales, Kinshasa, République Démocratique du Congo; 2 Service de Microbiologie, Cliniques Universitaires de Kinshasa, Faculté de Médecine, Université de Kinshasa, République Démocratique du Congo; 3 Direction de la Lutte contre la Maladie, Ministère de la Santé de la République Démocratique du Congo; 4 Ecole de Santé Publique de l’UNIKIN, République Démocratique du Congo

**Keywords:** Epidémie, grippe humaine, RDC, surveillance, Epidemic, human influenza, DRC, monitoring

## Abstract

En République Démocratique du Congo (RDC), plusieurs épidémies de la grippe restent méconnues car confondues à d'autres pathologies infectieuses avec lesquelles elle partage la même symptomatologie. L'objet de cette étude était de faire l'état de lieu des épidémies de grippe rapportée en RDC avant 2008, l'année de la mise en place de la surveillance de la grippe en RDC. Nous avons recherché tous les documents (articles, rapport,…) ayant rapporté une épidémie de grippe ou d'infection respiratoires aigues (IRA) en RDC avant 2008 en utilisant des mots clés choisis. Pour chaque rapport retrouvé, les éléments de description d'épidémie ont été recherchés et analysés. Quatre documents ont été retrouvés dont aucun article publié. Les sites des épidémies rapportées étaient les Zones de santé rurales de Koshibanda et Kahemba dans le Bandundu (1995 et 2007), de Bosobolo à l'Equateur (2002) et de Kinshasa (2002-2003). Les taux d'attaque et de létalité étaient respectivement de 3.9% et 16% à Koshibanda ; de 0.1% et 2% à Kinshasa ; de 47.5 % et 1.5% à Bosobolo et de 14.6 % et 2.9% à Kahemba. Les enfants de moins de 5 ans étaient les plus touchés. Leurs taux d'attaque variaient entre 22.6 et 57.7% et les taux de létalité entre 3.2 et 3.7%. Les deux épidémies de Bosobolo et de Kinshasa étaient associées au virus influenza H3N2.Cette revue montre une grande morbi-mortalité des rares épidémies de grippe rapportées en RDC.

## Abstract (en)

In the Democratic Republic of the Congo (DRC), several influenza epidemics are ignored because they are confused with other infectious diseases which have similar symptoms. Our study aims to assess influenza epidemics occurred in the DRC before 2008, year of the implementation of the influenza surveillance program in the DRC. We searched all the documents [articles, report,…] about influenza epidemic or acute respiratory infections [ARI] in the DRC before 2008 by using chosen key words. Epidemic description elements were identified and analyzed in each report. 4 documents have been found that had no article published. The sites of the epidemic outbreak were the rural health zones in Koshibanda and Kahemba, Bandundu [1995 and 2007], in Bosobolo, Equator [2002] and in Kinshasa [2002-2003]. Attack and lethality rates were 3.9% and 16% in Koshibanda respectively; 0.1% and 2% in Kinshasa; 47.5% and 1.5% in Bosobolo and 14.6% and 2.9% in Kahemba. Children less than 5 years of age were the most affected. Their attack rates ranged between 22.6 and 57.7% and lethality rates ranged between 3.2 and 3.7%. The two epidemics in Bosobolo and Kinshasa were associated with H3N2 influenza virus. This literature review highlights a high morbidity and mortality due to rare influenza epidemics in the DRC.

## Introduction

La grippe est une infection virale aiguë des voies respiratoires qui est très contagieuse, et caractérisée par la fièvre, des céphalées, des myalgies, des maux de gorge et la toux. Elle est causée par les virus influenza qui sont des virus à ARN, à génomes segmentés dont on distingue trois types A, B et C sur la base de l'antigénicité de leur nucléoprotéine [1, 2]. La grippe provoque des épidémies annuelles qui atteignent leur pic pendant l'hiver dans les régions tempérées [3]. Les virus responsables des épidémies (type A et B) présentent une grande plasticité, ce qui leur permet de modifier en permanence leurs caractéristiques antigéniques [4]. Les virus grippaux de type A, souvent à la base des pandémies, se subdivisent en sous-types en fonction de la nature et de l'association de leurs antigènes de surface représentés par l'hémagglutinine (H) et la neuraminidase (N). Parmi les nombreux sous-types des virus grippaux A, les sous-types A (H1N1) et A (H3N2) circulent actuellement chez l'homme et sont responsables de la pathologie humaine [5]. Par contre le virus B, essentiellement infectieux pour l'homme, est stable et comprend deux lignées génétiques différentes nommées en fonction des régions où elles ont été identifiées pour la première fois: l'influenza B de la lignée Victoria et l'influenza B de la lignée Yamagata [6]. Les virus de la grippe saisonnière circulent chaque année et causent la maladie chez les humains. Dans les pays à climats tempérés, la maladie tend à se produire de façon saisonnière sous forme d'épidémie d'ampleur variable principalement au cours de l'hiver [7] ; la propagation de la maladie ne s'y fait de personne à personne que par les éternuements, la toux ou le contact avec des surfaces contaminées. Ces infections peuvent causer des formes variées de maladies allant d'une légère à une grave maladie, voire la mort, en particulier chez certains individus à haut risque constituant ainsi de ce fait un problème de santé publique sérieux pour lequel on dénombre chaque année dans le monde 3 à 5 millions de cas, dont 250000-500000 sont mortels [8] Elle représente une cause importante de morbidité élevée et de mortalité chez les enfants et les personnes âgées [9] essentiellement dans les régions tempérées d'Europe [10] et d'Amérique [11]. Dans les régions tropicales, les épidémies de grippe sont très peu documentées [12]. La grippe y est mal connue par le corps médical qui la confond avec les affections courantes comme le paludisme et la fièvre typhoïde [2, 13]. En République Démocratique du Congo (RDC), plusieurs épidémies de la grippe restent méconnues car confondues à d'autres pathologies comme la malaria et la fièvre typhoïde avec lesquelles elle partage la même symptomatologie. Cette confusion entraine d'une part une mauvaise prise en charge de cette maladie aux conséquences graves et d'autre part, prive les décideurs de données sur le véritable poids de cette maladie devant aider à la mise en place des mesures de lutte contre cette maladie. Cependant, en 2006, suite à la menace de la pandémie de grippe due à l'émergence du virus influenza aviaire H5N1 depuis 2003, accompagnée de plusieurs cas humains signalés dans la région d'Afrique, des efforts ont été fournis par les gouvernements des pays appuyés par le CDC, l'OMS et d'autres organismes internationaux pour mettre en place des systèmes de surveillance de la grippe en Afrique [8]. En RDC, un système de surveillance sentinelle et national de la grippe a été mis en place par le ministère de la santé appuyé par le CDC/Atlanta et l'OMS. L'objet de cette étude était de faire l'état de lieu des épidémies de grippe rapportée en RDC avant la mise en place de la surveillance de la grippe dans le pays.

## Méthodes

**Type et cadre de l'étude :** il s'agit d'une étude rétrospective documentaire basée sur des articles et des rapports d'épidémies de grippe et des IRA en RD Congo. Nous avons recherché les documents ayant trait aux épidémies de la grippe et des virus respiratoires aigues (IRA) ayant eu lieu avant 2008, l'année de la mise en place de la surveillance de la grippe en RDC. La recherche des articles a été faite en utilisant différents moteurs de recherche (Pubmed, Google,…). Les mots clés suivants : « épidémie, grippe, RD Congo, infection respiratoire aigue » en français ou encore « outbreak, influenza, DR Congo, acute respira tory infections » en anglais ont été employés. Les rapports ont été recherchés dans les archives de la direction de la lutte contre la maladie du Ministère de la Santé Publique de la RD Congo, de l'Institut National de Recherche Biomédicale (INRB), de la Faculté de Médecine de l'Université de Kinshasa ainsi que dans ceux des certains ONG internationales telles que MSF, OMS,… [14–17].

**Collecte des données:** pour chaque document obtenu, des informations suivantes ont été collectées à l'aide d'une fiche de collecte préétablie. Il s'agissait des paramètres suivants : année de publication, date alerte de l'épidémie, semaines de début, semaine du pic épidémique, lieux ou sites investigués, la définition des cas utilisés, population affectée, le taux d'attaque et le taux de létalité, type de prélèvement, le lieu de diagnostic, méthode diagnostique utilisée, type et sous type de virus en cause.

**Analyses de données:** toutes les données collectées étaient saisies à l'aide du logiciel Excel, Microsoft office 2007. L'analyse de base, la production des tableaux et des figures avaient été effectuées avec le logiciel Excel, Microsoft office 2007.

**Principales caractéristiques des épidémies de grippe en RDC avant 2008 :** au total 3 rapports d'investigations d'épidémies ont été retenus ainsi qu'un bulletin épidémiologique mensuel des maladies à potentiel épidémique. Aucun article publié n'a été retrouvé. Ces rapports ont été faits essentiellement par les ONG tel que le MSF, par la direction de la lutte contre la maladie et par l'inspection médicale urbaine de Kikwit dans la province de Bandundu en RDC. Ces rapports concernaient 2 zones de santé rurale (ZSR) dans le Bandundu, une ZSR à l'Equateur et plusieurs zones de santé de la ville province de Kinshasa .Ces épidémies ont été rapportées à Koshibanda (1995), à Bosobolo (2002), à Kinshasa (2003) et à Kahemba (2007). Les taux d'attaque et de létalité étaient respectivement de 3.9% et 16% à Koshibanda ; de 0.1% et 2% à Kinshasa ; de 47.5 % et 1.5% à Bosobolo et de 14.6 % et 2.9% à Kahemba. Les populations investiguées étaient respectivement de 2620 habitants à Koshibanda repartis sur 8 villages, 1145 à Kahemba repartis sur 3 villages ,2629 à Bosobolo repartis sur 8 villages et 5268736 habitants à Kinshasa repartis sur les 24 communes de la ville province de Kinshasa. Toutes les épidémies rapportées avaient débuté entre la semaine 35 et la Semaine 41 sauf celle de Kahemba qui avait commencé à la semaine 17. Le Tableau 1 montre les principales caractéristiques des épidémies dans les différents sites. Les enfants de moins de 4 ans étaient les plus affectés avec des taux de létalité les plus élevés de 3.2% à Kinshasa et de 3.5% à Bosobolo ; suivis des personnes âgées de plus de 65 ans à Bosobolo et de jeunes de 5-14ans à Kinshasa avec des taux de létalité respectifs de 3.2% et de 1.7%. Le Tableau 1 montre la répartition des cas et des décès par tranche d'âge dans les sites investigués.

**Tableau 1:** Caractéristiques générales des épidemies par sites

Paramètres	ZS Bosobolo	ZS Kinshasa	ZS Kahemba	ZS Koshibanda
Année de Survenue	2002	2002	2007	1995
Semaine de début	S35	S41	S17	S36
Semaine du pic	S44	S2/2003	S21	S45
Nombre de Sites affectés	8 villages	24 communes	3 villages	8 villages
Population investiguées	2629 habitants	5268736 habitants	1145 habitants	2620 habitants
Alerte de l’épidémie	16 Nov. 2002	Déc. 2002	05-juin-07	Nov. 1995
Définition des cas utilisés	Définition OMS^+^	Définition l’OMS^+^	Définition OMS^+^	Définition OMS^+^
Nombre de cas	1245	7357	168	645
Nombre décès	18	146	5	101
Taux d’attaque^+^	47.4%	0.1%	14.6%	28.4%
Taux de létalité^++^	1.5%	2%	2.9%	16%
Tranche d'age affectée	<4ans	<4ans	<4ans	-
<4ans	252 cas (20%)	3502 cas (47,6%)	38 cas (22,6%)	
5-14ans	308 cas (24,7%)	20 cas (0,2%)	29 cas (17,2%)	
>65ans	60 cas (4,8%)	99 cas (1,3%)	-	
**Taux de létalité par tranche d'âge**				
<4ans	3,50%	3,20%	0,60%	
5-14ans	0,30%	1,70%	0%	
>65ans	3,20%	1%	-	

OMS=une personne présentant de manière brutale une fièvre élevée, des myalgies et/ou des céphalées, et un syndrome respiratoire (toux, mal de gorge et/ou rhinorrhée)

++ Taux de Létalité=Nb décès /Nb de cas

+ Taux d’Attaque=Nb cas/population investiguée

**Diagnostique et étiologies des Infections respiratoires aigües :** sur les 4 épidémies ayant été rapportées, 3 ont fait l'objet d'analyse de laboratoire pour déterminer les étiologies. La souche virale H3N2 a été retrouvée lors des épidémies de Bosobolo et de Kinshasa alors qu'à Kahemba, c'est la souche virale RSV qui avait été retrouvée avec quelques souches du virus influenza A/H3N2 co-circulant. Le Tableau 1 détaille les éléments de diagnostic et les virus responsables.

## Etat actuel des connaissances

La connaissance de l'impact de la grippe est importante à savoir afin de mettre en place des strategies de lutte adequate particulierement dans le pays en developement où l'ampleur de cette maladie est meconnu. L'objectif de cette étude était de faire la revue des epidemies de grippe humaine enregistrée en RDC avant la mise en place de la surveillance nationale. Nous avons montré une faible documentation des epidemies de la grippe en RDC, la rareté des données sur les epidemies de grippes en RDC. Cependant ces rares données traduisent une grande morbimortalité.

**Rareté des données sur la grippe en RDC:** avant 2008, seulement 4 documents ont rapportés des données sur les infections respiratoires aigues dues à la grippe en RDC traduisant ainsi une rareté de ces données avant la mise en place du système national de surveillance de la grippe. Ce constat est également fait par des nombreuse études en Afrique avec comme conséquence la sous estimation de l'impact de la grippe dans les pays d'Afrique subsaharienne en général et en RDC en particulier [12]. En effet une revue sur la grippe publiée en 2010 montrait que les données sur la grippe étaient insuffisantes en Afrique subsaharienne pour prioriser les stratégies de prévention et de contrôle en Afrique. Cette rareté s'explique par le fait que la surveillance de la grippe n'était pas courante en Afrique avant la menace de la pandémie grippale due au virus aviaire H5N1 en 2007 par manque d'infrastructure de laboratoire pour la confirmation de cas et par le manque d'intérêt des programmes de lutte contre les maladies étant donné que la grippe était considérée comme sévissant uniquement dans les régions froides. Les seuls pays d'Afrique qui avaient déjà initié cette activité avec des résultats efficaces sur la morbi-mortalité étaient l'Afrique du sud, le Madagascar et le Sénégal ; alors que les autres pays de l'Afrique subsaharienne n'ont installé leur système de surveillance qu'à la suite de l'émergence du virus aviaire H5N1 depuis 2003. En outre, la maladie est cliniquement semblable à des affections les plus fréquentes comme le paludisme et la fièvre typhoïde, et, est souvent diagnostiquée et traitée comme telles sans confirmation de laboratoire. Les données disponibles étaient en fait l'effort des organismes comme les MSF qui intervenait lors des épidémies afin d'établir les causes étiologiques probable et /ou confirmée.

**Morbidité et mortalité de la grippe en RDC avant la mise en place de la surveillance nationale:** malgré leur rareté, ces données font état d'une morbi-mortalité due à la grippe relativement élevée chez les jeunes enfants de moins de 5ans et aussi chez les personnes âgées de plus de 65 ans. Des taux de létalité étaient également élevés chez les personnes âgées de plus de 65 ans, particulièrement à Bosobolo avec 3.2% et à Kinshasa avec 1% mais l'impact général de la grippe dans cette tranche d'âge était difficile à évaluer à cause du faible nombre de personnes de cette tranche d'âge à consulter des structures de santé dans notre milieu ; et aussi espérance de vie qui est faible dans notre milieu, rendant cette tranche d'âge minoritaire, par rapport aux pays du nord où la majorité de la population est âgée. Des études similaires réalisées en Afrique ont noté que la grippe est communément identifiée chez les jeunes enfants qui souffrent d'infection respiratoire aigue [18–21]. De même que dans les pays des régions de l'hémisphère nord, les jeunes enfants payent le lourd fardeau des infections respiratoires et des hospitalisations dues à la grippe [22], similaire à ce que nous avons trouvés dans notre milieu. Des taux élevés de morbidité et de létalité trouvés à Koshibanda, à Kinshasa et à Bosobolo témoignaient de la gravité de la maladie. Et ces taux étaient comparables à ceux trouvés à Madagascar en Aout de la même année, peu avant la survenue des épidémies de Bosobolo et de Kinshasa, lors d'une épidémie d'infection respiratoire d'origine grippale survenue à Madagascar en 2002 [23]. A Kahemba par contre, l'épidémie aurait débuté pendant la saison sèche, à la semaine S17 suivie d'une augmentation progressive des cas d'IRA atteignant leur pic à la semaine S21 de l'année 2007 soit de mai à juin 2007. Ceci pourrait s'expliquer par le fait que les étiologies de ces épidémies étaient différentes ; celle de Kahemba étant due principalement au Virus Respiratoire Syncycial (RSV). La circulation du virus influenza durant cette période était sporadique. Bien que non confirmée biologiquement, l'épidémie de Koshibanda dans le Bandundu avait commencé vers le mois de septembre et avait atteint son pic au mois de novembre 1995. Par sa létalité (16%) et la période de sa survenue, cette épidémie serait comparable aux épidémies de Bosobolo et de Kinshasa causée par le virus H3N2 en saison de pluie qui est habituellement accompagnées de fortes humidités sous les tropiques [24].

**Souche responsable de l'épidémie:** le virus influenza A/H3N2 était associé à toutes les épidémies rapportées dans cette revue. Cette souche était proche du variant du virus influenza A/H3N2 /Moscow/10/99 qui a été décrit dans la composition du vaccin antigrippale de la saison 2002 de l'hémisphère sud et de la saison 2002-2003 de l'hémisphère nord. Les virus grippaux A/H3N2 étaient associées à des flambées dans de nombreux pays d'Europe, d'Amérique et d'Afrique en 2002 ; la plupart d'entr'eux étaient comparables au virus A/Moscow/10/99 et à la souche vaccinale largement utilisée, A/Panama/2007/99 [25]. Le Tableau 2 montre la répartition des souches virales responsables des épidémies rapportées dans les différents sites investigués.

**Tableau 2:** Caractéristiques Etiologique des épidemies par sites

Paramètres	ZS Bosobolo	ZS Kinshasa	ZS Kahemba	ZS Koshibanda
Lieu de la confirmation du diagnostic	Institut Pasteur de Paris	Institut Pasteur de Paris	Laboratoire national de santé de Luxembourg	-
Type de prélèvement	Sérum et sang total ; frottis nasopharyngés+MTV	Sérum et sang total ; frottis nasopharyngés+MTV	Frottis nasopharyngés et pharyngés +MTV	-
Méthode diagnostique	Sérologie et Culture	Sérologie et Culture	RT-PCR	Clinique
Sous type	H3N2^^+^^	H3N2^+^	RSV, H3N2^+^	H3N2

+ =H3N2 (A/Moscow/10/99(H3N2)

## Conclusion

cette revue montre que les données sur les épidémies de grippes en RDC avant la mise en place d'un système de surveillance nationale de grippe sont rares. Cependant, elles s'accompagnent d'une grande morbi-mortalité. La mise en place d'un système de surveillance est donc vivement souhaitée pour mieux estimer l'impact de cette maladie en RDC.

### Etat des connaissances actuelles sur le sujet

- Absence de données sur l'ampleur de l'épidémie de grippe en RDC.

### Contribution de notre étude à la connaissance

- Les données parcellaires obtenues de cette revue suggèrent que la grippe est présente en RDC ;
- La grippe pourrait être une cause importante de maladies respiratoires, particulièrement chez les enfants ;
- Le renforcement de la surveillance de la grippe déjà en place depuis 2008 est indispensable pour évaluer l'impact de la grippe et estimer sa saisonnalité en RDC.

## Conflits d’intérêts

Les auteurs ne déclarent aucun conflits d'intérêt.
